# Spectroscopic Study of Strain, Oxidation, and Formation of Few-Layer Graphene at Steel Surfaces Tribologically Tested against MoS_2_ Films

**DOI:** 10.1021/acsami.4c11016

**Published:** 2024-09-26

**Authors:** A. Wittrock, A. Wittig, C. A. Thomann, D. Stangier, N. F. Lopes Dias, W. Tillmann, J. Debus

**Affiliations:** † Department of Physics, TU Dortmund University, 44227 Dortmund, Germany; ‡ Institute of Materials Engineering, TU Dortmund University, 44227 Dortmund, Germany

**Keywords:** tribology, MoS_2_, transfer material, strain, oxidation, graphene, Raman spectroscopy

## Abstract

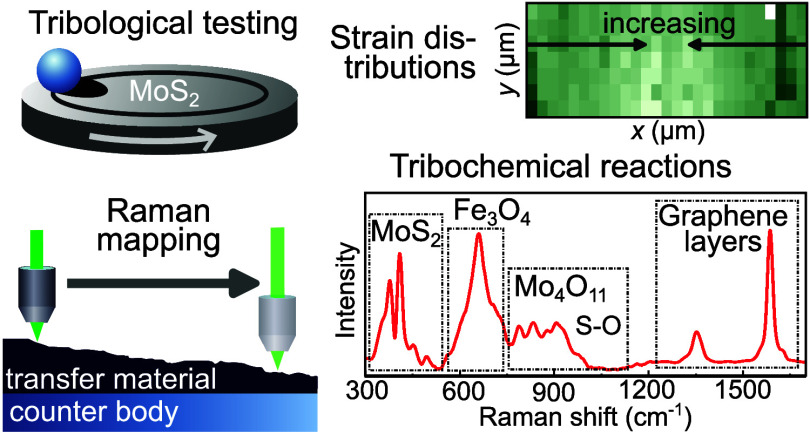

MoS_2_ not only has unique optoelectronic properties realizing photonic and semiconductor applications but also serves as a promising solid lubricant in tribological three-body contacts due to its advantageous friction and wear behavior. Its functionality is defined by elementary processes including strain, oxidation processes, and material mixing. However, these mechanisms were not elucidated for MoS_2_ having transferred from the MoS_2_ film synthesized at the main body to a steel counter body during tribological ball-on-disk tests. Using spatially and spectrally high-resolved Raman spectroscopy, we study the compressive and tensile strain within the MoS_2_ transfer material and analyze the oxidation of molybdenum, sulfur, and iron. In addition, we elaborate on the impact of transition metals modifying the MoS_2_ films on the strain distribution and oxidation processes. Decreasing intensities of the MoS_2_ Raman lines are accompanied with enhanced intensities of sulphur and molybdenum oxide Raman signatures which are particularly agglomerated at the edges of the tribological track. The formation of tribochemical oxides, including Mo_4_O_11_ in the Magnéli-phase, depends weakly on the type of modifying element, and an oxidation of a modification element itself is not detected. We also identified a tribologically induced formation of disordered few-layer graphene at counter-body surface areas which experienced weak thermo-mechanical tribological load. Our results characterize structural and chemical features of the MoS_2_ transfer material, thus predicting material failure at the microscopic level.

## Introduction

Transition metal dichalcogenides (TMDCs) are used in diverse fields of research. TMDC monolayers, which consist of one layer of transition metal atoms embedded between two layers of chalcogen atoms bound to each other by van-der-Waals interactions, possess unique optoelectronic and magneto-optical properties being attractive for semiconducting physics including spin- and valleytronics.^1−3^ Micrometer-thin layers of TMDCs with a lamellar structure are moreover exploited as solid lubricants in complex dry sliding tribological contacts in which the individual atomically thin layers easily slide against each other. Among the application of TMDCs as solid lubricants, MoS_2_ attracts particular attention.^4,5^ The tribological behavior of MoS_2_ highly depends on the load and wear conditions including the pressure, humidity, and chemical and physical structures of the synthesized MoS_2_ films.^6,7^ Especially, normal (humid) environments enhance the friction due to the adsorption and diffusion of water molecules leading to large shear resistance.^8,9^

To further improve the tribological properties of MoS_2_, especially under normal conditions, the films are modified by high chemical element doping.^10,11^ The modification elements are integrated into the layered structure of MoS_2_ in different ways: The modification atoms may substitute Mo or S atoms (e.g., W^12^), they may occupy interstitial sites (e.g., Cu^10^), or they may be intercalated between the layers (e.g., V^13^). The way a modification element is integrated into the MoS_2_ lattice depends on the element itself, its concentration, and the synthetization process. Particularly promising seems to be the modification of MoS_2_ by transition metal X = Cr, Cu, V, or Zr. For instance, it was shown that the elements Cr or Zr may significantly improve the tribological properties, as they lead to a densification of the microstructure, which in turn results in a weak diffusion of water molecules into the film and thus a reduction in friction.^14−16^ However, increasing the amount of the modification element may cause a structural transformation from bulk crystalline to nanocrystalline and amorphous. Thus, after exceeding a critical amount of the incorporated element, the tribological properties are impaired. Furthermore, the presence of a modification element may initiate the formation of nucleation sites or, respectively, barriers favoring a crystallite growth with (002) basal-plane orientation.^17−19^ Although these studies ascribed the improved tribological features to changes in the microstructure and crystallite orientation, the interactions between the mechanisms determining the wear and friction of element-modified MoS_2_:X films are subject to ongoing research.

One of the key processes in especially lubricant-free tribological experiments with a main body sliding against a counter body is the transfer of material from the main to the counter body under normal conditions.^14^ In the following, we consider a main body with a surface made from MoS_2_:X and an uncoated counter body made from case-hardened steel (AISI 52100), which describes in combination with the environment the tribological system. We define transfer material as material transferred from the main to the counter body (and vice versa) potentially experiencing changes in structural and chemical properties. It contributes significantly to the tribological interaction between the main and counter body as well as the environment. Terminologically, transfer material belongs to the tribological material, which is formed due to friction and wear. Tribological material in turn results from chemical and physical interactions including, e.g., temperature- and stress-induced changes in the material composition. In this respect, oxide and sulfide compounds are typically created.^5,20^ Furthermore, small-sized wear particles may be formed during the tribological load, which may exhibit different friction and wear behavior including a variation in the chemical reactivity, strain distribution, or crystalline degree.^21−23^ These particles might be conceptually distinct from tribological material, since they may have been driven outside of the tribological contact, while tribological material is actively present within the contact area between the main and counter body.

So far, detailed studies on the spatial distribution of the tribological material at the counter body worn against MoS_2_ films and its structural and chemical features are missing. In this context, it is an open question how a transition metal X in MoS_2_:X may affect the transfer of material from the main body to the counter body. Hereby, it is interesting to specify the kinds of oxides that are formed and to identify their formation process as well as their influence on the transfer material. It is of great interest to gain knowledge about tribologically induced chemical reactions and the distribution of strain within the MoS_2_ transfer material. The detection of surficial mixed material with structural features differing from that of the substrate or initial surface material is also a challenging task. Hereby, specifying the role of carbon in/at the steel counter bodies is essential, since different kinds of tribologically beneficial carbon allotropes may be formed due to the thermal and mechanical conditions within the tribological contact. In the following, we provide answers to these questions and aspects for the transfer material at initially uncoated steel spheres (counter bodies) using confocal Raman scattering spectroscopy. In tribological ball-on-disk experiments, the counter bodies were tested against MoS_2_:X (X = Cr, Cu, V, or Zr) films (main bodies).

## Materials and Methods

The MoS_2_:X films were synthesized by magnetron sputtering (CC800/9 Custom, CemeCon AG, Germany) on top of a 16MnCr5 steel (AISI 5115) substrate having an average roughness value of (3.1 ± 0.6)nm and a hardness of (52 ± 1)HRC. The sputter targets consisted of two MoS_2_ targets and one target of the modifying element X = Cr, Cu, V, or Zr. Additionally, MoS_2_:Ti and MoS_2_:W films are analyzed; since the respective results are similar to that obtained for MoS_2_:Cr, they are not shown explicitly. The purity of the target materials used was about 99.9 at. %. Possible oxides and contaminants at the target and substrate surfaces were removed by heating and etching before the deposition process. For synthesizing an element-modified MoS_2_ film, the three targets were simultaneously sputtered; whereby, the MoS_2_ targets were sputtered in High-Power-Impulse Magnetron Sputtering (HiPIMS) mode with an average cathode power of 3 kW, a pulse frequency of 1 kHz, and a pulse duration of 200 μs. For the target of the modifying element, a direct current power supply was chosen with an adjusted power to obtain an amount of 20 at. % of the modification element within the films. As deposition parameters, a bias voltage of −100 V, an argon pressure of 400 mPa, and a heating power of 1 kW were selected. Further details on the synthesis process can be found in ref (24). Besides, the initial chemical composition of the thin films having a thickness between 2 and 2.5 μm was evaluated by energy-dispersive X-ray spectroscopy, which determines the S/Mo ratio between 1.6 and 2.3. Although the element-modified thin films are not perfectly stoichiometric, we use the designation MoS_2_ in the following taking into account that the mean value of the S/Mo ratio is about 2 and the Raman signals measured are unambiguously attributed to MoS_2_.

**Figure 1:**
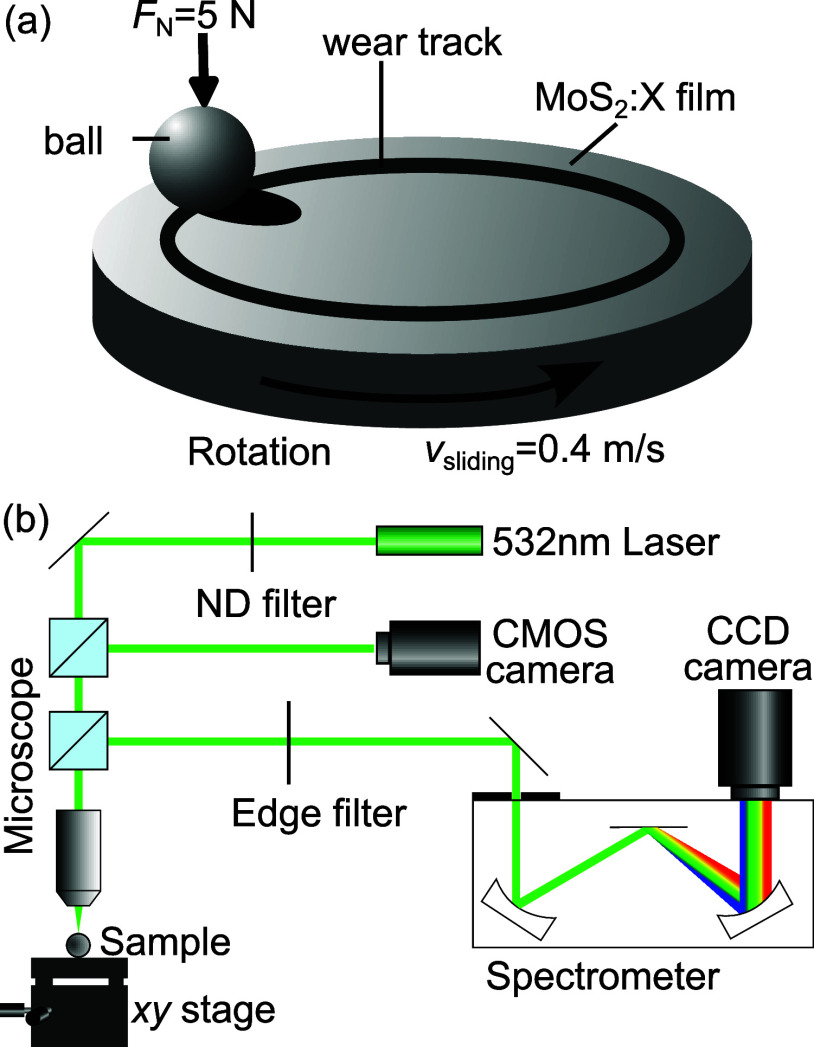
(a) Scheme of the tribological ball-on-disk setup showing the MoS_2_:X film (main body) and the steel sphere (counter body). (b) Scheme of the Raman scattering setup; the laser light is attenuated by a neutral density (ND) filter. A monochrome CMOS camera obtained an image of the sample area, including the laser spot position. Further details are given in the main text.

The MoS_2_:X films were tribologically tested under ambient conditions by using a ball-on-disk tribometer (CSM-Instruments, Switzerland). The ball-on-disk tribometer setup is sketched in Figure 1(a). The main bodies were the MoS_2_:X films on top of the steel substrates, while the counter bodies were case-hardened 100Cr6 steel (AISI 52100) spheres with (11.3 ± 0.2) GPa hardness. Main and counter bodies were tested against each other for 5000 circular rotations, a normal force of 5 N, and a constant sliding speed of 0.4 m/s. The morphology and the topography of the tribologically tested MoS_2_:X thin films are examined with a scanning electron microscope (JSM-7001 M JEOL, Japan).

The tribological tests were followed by nonpolarized Raman scattering measurements at the tribological contact area (wear track) of the main and, in particular, the counter body. A scheme of the confocal Raman scattering setup is presented in Figure 1(b). The optical excitation was realized by a 532 nm single-frequency laser whose power was set to approximately 1.5 mW, measured at the sample position. This excitation wavelength avoids resonant effects in MoS_2_ and possible tribochemical compounds like Mo oxides, as reported in refs (25,26). The laser light was focused onto the counter or main body using a microscope objective with 20× magnification, yielding a laser spot of about 2 μm diameter. For a spatial Raman scattering mapping, the sample was moved along the lateral *x* and *y* directions by a high-precision motorized stage. The Raman (inelastically) scattered light which was collected through the aforementioned microscope objective was finally analyzed by a 0.75 m focal-length spectrometer equipped with a 600 grooves/mm grating and a liquid-nitrogen-cooled charged-coupled-device (CCD) camera. It consisted of a front-illuminated Si chip with 2048 × 512 pixel area. The elastically scattered light was suppressed with a spectral edge filter.

## Results and Discussion

### Raman Scattering Spectra at the Main and Counter Body

**Figure 2:**
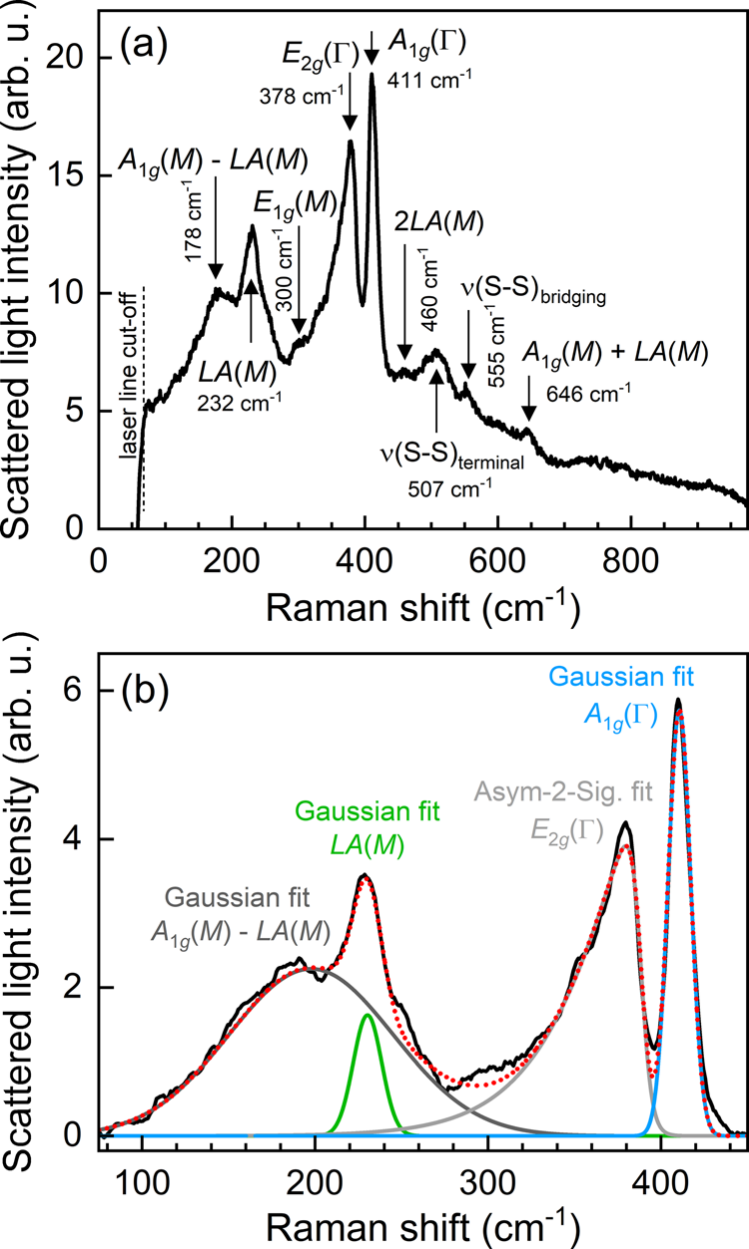
(a) Exemplary Raman scattering spectrum of MoS_2_ excited with laser light of 532 nm wavelength; the characteristic phonon modes of MoS_2_ are assigned. (b) Exemplary fit curves for modeling the phonon modes A_1g_(M) – LA(M), LA(M), E_2g_(Γ), and A_1g_(Γ). The total fit function is displayed by the red dotted curve.

The spectrum shown in Figure 2(a) demonstrates the characteristic Raman scattering lines measured both at the main body and within the MoS_2_ transfer material at the counter body. It contains intense Raman bands at about 178, 232, 378, and 411 cm^–1^ which are based on the Raman-active acoustic and optical phonon modes A_1g_(M) – LA(M), LA(M), E_2g_(Γ), and A_1g_(Γ) of multiple monolayer-thick and/or bulk-like MoS_2_, respectively.^27^ The E_2g_ mode corresponds to the in-plane vibrations of two sulfur atoms, with respect to a molybdenum atom. The out-of-plane vibrations of sulfur atoms in opposite directions are the origin of the A_1g_ mode. Both modes are first-order modes at the center Γ of the Brillouin zone. In the Raman spectra, phonon modes A_1g_(M) – LA(M) (178 cm^–1^) and LA(M) (232 cm^–1^) are also prominent. The longitudinal acoustic phonons LA(M) at the *M*-point of the Brillouin zone are in-plane collective movements of all atoms.

In the following, mainly the LA(M) mode as well as the optical vibrational modes E_2g_(Γ) and A_1g_(Γ) are studied (Γ will no longer be mentioned), as their signal-to-noise ratios are pronounced and their line parameters are useful to evaluate structural changes within the MoS_2_ structure. While the LA(M) and A_1g_ Raman lines are well described by Gaussian functions, the asymmetric E_2g_ line is fitted by an asymmetric double sigmoidal function. For stabilizing the fitting routine of the LA(M) line, additionally the A_1g_(M) – LA(M) band is approximated by a Gaussian function, as shown in Figure 2(b). The intensity, spectral position, and width of the Raman lines are of central interest for assessing the structural properties of MoS_2_:X. For evaluating the presence of MoS_2_ at the surfaces studied, we take into account the ratio between the Raman line intensities *I*[A_1g_] and *I*[E_2g_]: *I*_R_[MoS_2_] = *I*[A_1g_]/*I*[E_2g_]. In this way, the contributions of both the central out-of-plane and in-plane lattice vibrations are considered. Moreover, it is known—in particular from MoS_2_ atomic monolayer and few-layer structures—that (i) the intensity ratio decreases for heavily damaged and defective MoS_2_, (ii) strain reduces slightly the intensity ratio, and (iii) out-of-plane oriented grains lead to a significant enhancement of *I*_R_[MoS_2_].^28^ Accordingly, the intensity ratio allows for qualitative assessment of the structural properties of the MoS_2_:X films and transfer material.

**Figure 3:**
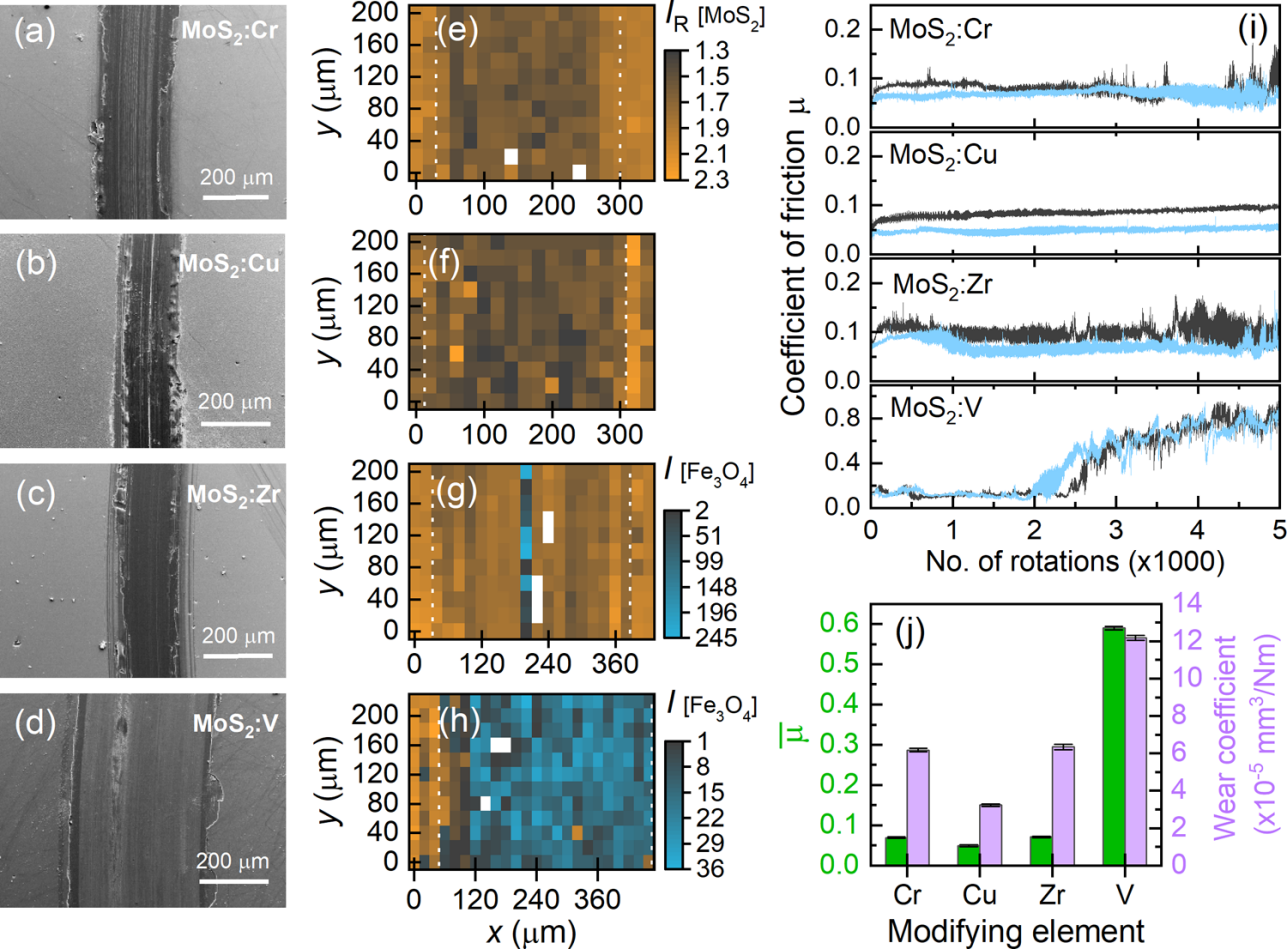
(a)–(d) SEM images and (e)–(h) Raman mappings of tribological track areas for the differently element-modified MoS_2_:X films. The Raman mappings show the intensity ratio *I*_R_ and the Raman scattering intensity of the Fe_3_O_4_-related A_1g_ mode at about 661 cm^–1^. The dashed vertical lines indicate the edges of the tribological track. (i) Coefficient of friction as a function of the number of ball-on-disk rotations, for the different MoS_2_:X films and the 10 mm (dark-gray curves) and 12 mm (light-blue curves) radius of the wear track. (j) Average coefficient of friction μ̅ (left scale) and wear coefficient (right scale) compared for the element-modified films (main body). The errors represent the standard deviation of the average friction coefficient measured over a distance of 5000 rotations and, respectively, of the wear coefficient obtained from three measurements for each element-modified MoS_2_ film.

Scanning electron microscope (SEM) images of the tribological tracks at the main bodies with MoS_2_:X (X = Cr, Cu, Zr or V) surfaces are depicted in Figure 3(a)–(d). The track (dark area) results from the tribological (ball-on-disk) contact between the plane main body and spherical counter body, as described in the previous section. The bright areas in the images indicate unworn MoS_2_:X films. The widths of the tribological tracks lie within a range of 200 μm with the exception of the track at the MoS_2_:V surface which is broadened to approximately 400 μm.

Raman scattering mappings across the tribological tracks of the main body are shown in Figure 3(e)–(h). The intensity ratio *I*_R_[MoS_2_] is clearly reduced down to 1.3 within the track, outside of it *I*_R_[MoS_2_] increases above 2. The reduced intensity ratio *I*_R_[MoS_2_] within the tribological track indicates structural degradation of the MoS_2_ film, particularly due to increased strain and a realignment of grains with an in-plane orientation. Additionally, a peculiarity is observed for the MoS_2_:Zr and MoS_2_:V films; see panels (g) and (h): Within the tribological track, Raman scattering signals of magnetite (Fe_3_O_4_) are detected instead of MoS_2_ Raman lines. For MoS_2_:Zr, Fe_3_O_4_ is observed only at the center of the track, while, for MoS_2_:V, the complete tribological track exhibits Fe_3_O_4_ Raman scattering signatures. Although details will be given in the following, it is already obvious that the vanadium-modified MoS_2_ film was significantly removed during the ball-on-disk tests.

In Figure 3(i), the coefficient of friction μ is depicted as a function of the ball-on-disk rotation number, for the different combinations of MoS_2_:X main bodies and steel spheres. Using a different radius of the wear track of 10 mm (dark-gray curves) and 12 mm (light-blue curves) the coefficient of friction lies about 0.1 during the tests, only for MoS_2_:V, it increases significantly after 2000 rotations. In the latter case, the resulting values for the coefficient of friction μ are typical for a sliding contact between the steel surfaces. The arithmetic mean value μ̅ of the coefficient of friction is given in Figure 3(j) underlying the frictional behavior described in the framework of panel (i). Thus, the modified films exhibit slightly lower friction than nonmodified MoS_2_ films whose average coefficient of friction amounts to μ̅ = 0.15 ± 0.01.^24^ Moreover, the lowest wear coefficient of about 3 × 10^–5^ mm^3^/Nm, see the right scale in Figure 3(j), is measured for the MoS_2_:Cu film which is half the value evaluated for the MoS_2_:Cr and MoS_2_:Zr films. The highest wear coefficient belongs to the MoS_2_:V film which significantly exceeds the wear coefficient of (7.7 ± 0.1) × 10^–5^mm^3^/Nm of a nonmodified MoS_2_ film.^24^

In general, the friction behavior of the solid-state lubricant MoS_2_ is governed by the easy shearing of the S–Mo–S layers due to the weak van-der-Waals forces between the layers. The incorporation of a modifying element into the van-der-Waals gap may lead to a spatial lattice extension along the (100) edge-plane direction.^29,30^ This reduces the attractive force between the neighboring S planes, providing enhanced shearing and frictional properties. Furthermore, the element modification of MoS_2_ films may give rise to a (002) basal-plane orientation and densification of the microstructure.^31,32^ It also makes the incorporation of water molecules into the film less probable,^8^ thus contributing to low friction compared to nonmodified MoS_2_. The high coefficient of wear and loss of the MoS_2_:V film within the tribological track are assigned to the water adsorption capabilities of the polycrystalline vanadium.^33^ Additionally, due to an increased amount of water molecules, the wear particles agglomerate, become brittle, and leave the contact area more easily.^34^

**Figure 4:**
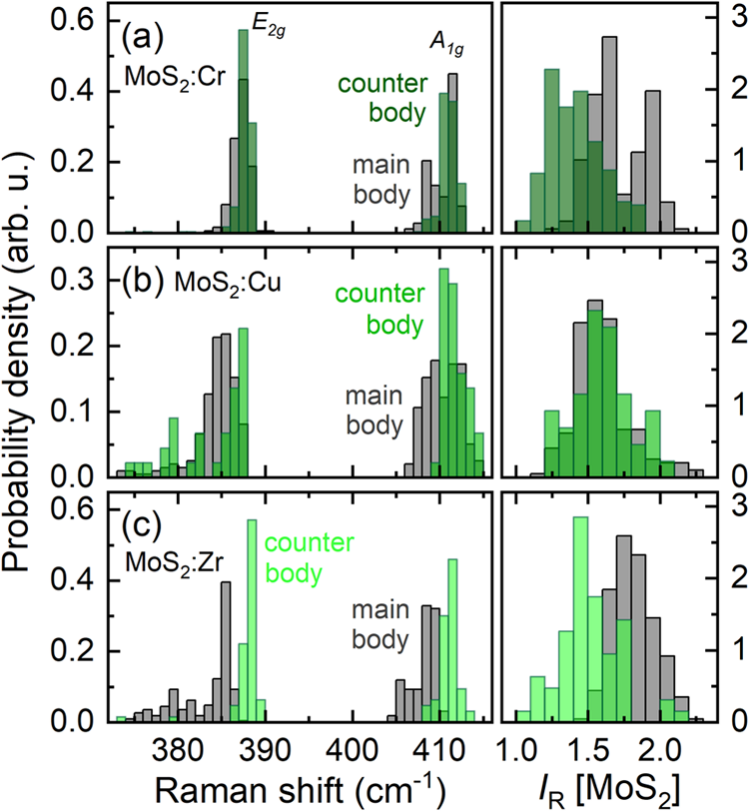
(a)–(c) Probability densities of the Raman shift values for the A_1g_ and E_2g_ lines and intensity ratio *I*_R_ detected within the transfer material at the counter bodies and the worn MoS_2_:X films of the main body. The values are based on at least 150 mapping points measured for each main and counter body. Note that the worn MoS_2_:V films, and even more so their counter bodies, show almost exclusively iron oxides after the tribological test, so they are not considered here.

Comparing the Raman spectra detected from the transfer material at the steel counter bodies with the spectra recorded within the tribological track of the main body, it becomes clear that both the spectral positions of the E_2g_ and A_1g_ Raman lines and their intensity ratios differ significantly from each other. The probability densities of the Raman shifts and intensity ratio *I*_R_, for the different main- and counter-body tribological combinations, are shown in Figure 4(a)–(c). In the case of MoS_2_:Cr depicted in Figure 4(a), the Raman shifts of the E_2g_ and A_1g_ modes detected at the main body lie within the same range as those measured at the tribological track of the counter body. Hence, one may conjecture that the MoS_2_:Cr at the worn main body and the MoS_2_:Cr transfer material at the counter body are similarly strained. The intensity ratio, for the counter body, is mainly below 1.5, while for the main body, it predominantly exceeds 1.5. This low *I*_R_ for the MoS_2_ transfer material underlines a structural degradation mainly originating from a realignment of grains from an out-of-plane to an in-plane orientation. A similar trend is also observed for the case of MoS_2_:Zr. For MoS_2_:Cu the intensity ratio values registered at the main and counter body are similarly distributed between 1 and 2.3 with a calculated average of about 1.55. Thus, the MoS_2_:Cu grains at the main body experienced a pronounced reorientation during the ball-on-disk test. The tribological impact on the MoS_2_:Cu film at the main body is also indicated by the quite broad distributions of the Raman shifts for both phonon modes. In particular, the out-of-plane vibration A_1g_ is more disturbed at the main body than at the counter body. This may be attributed to a cladding effect and an agglomeration of copper^35^ and its lubricating behavior owing to its low shear strength^36^ which both allow for structural realignments with various interaction strengths and atomic distances influencing the Raman shifts. These combined effects contribute to maintaining stable low friction and reduced wear for MoS_2_:Cu. A similar picture is presented in Figure 4(c), for MoS_2_:Zr; moreover, the Raman shifts of the E_2g_ line measured at the main and counter bodies’ tribological track strongly differ from each other. This discrepancy may be due to high tensile strain, which results in a symmetry breaking for the MoS_2_:Zr structure lifting the degeneracy of the E_2g_ mode. It in turn gives rise to a splitting into two subbands E_2g_^–^ and E_2g_^+^.^37,38^ On the whole, this splitting remarkably affects the peak position and leads to strong variations. Interestingly, the MoS_2_:Zr transfer material at the steel counter body does not exhibit this phenomenon. It is likely due to the absence of iron oxides at the counter body, while at the main body, at least, the center of the tribological contact is characterized by removal of the MoS_2_:Zr film, as discussed in the framework of Figure 3(g).

### Strain Distribution in MoS_2_ Transfer Material

**Figure 5:**
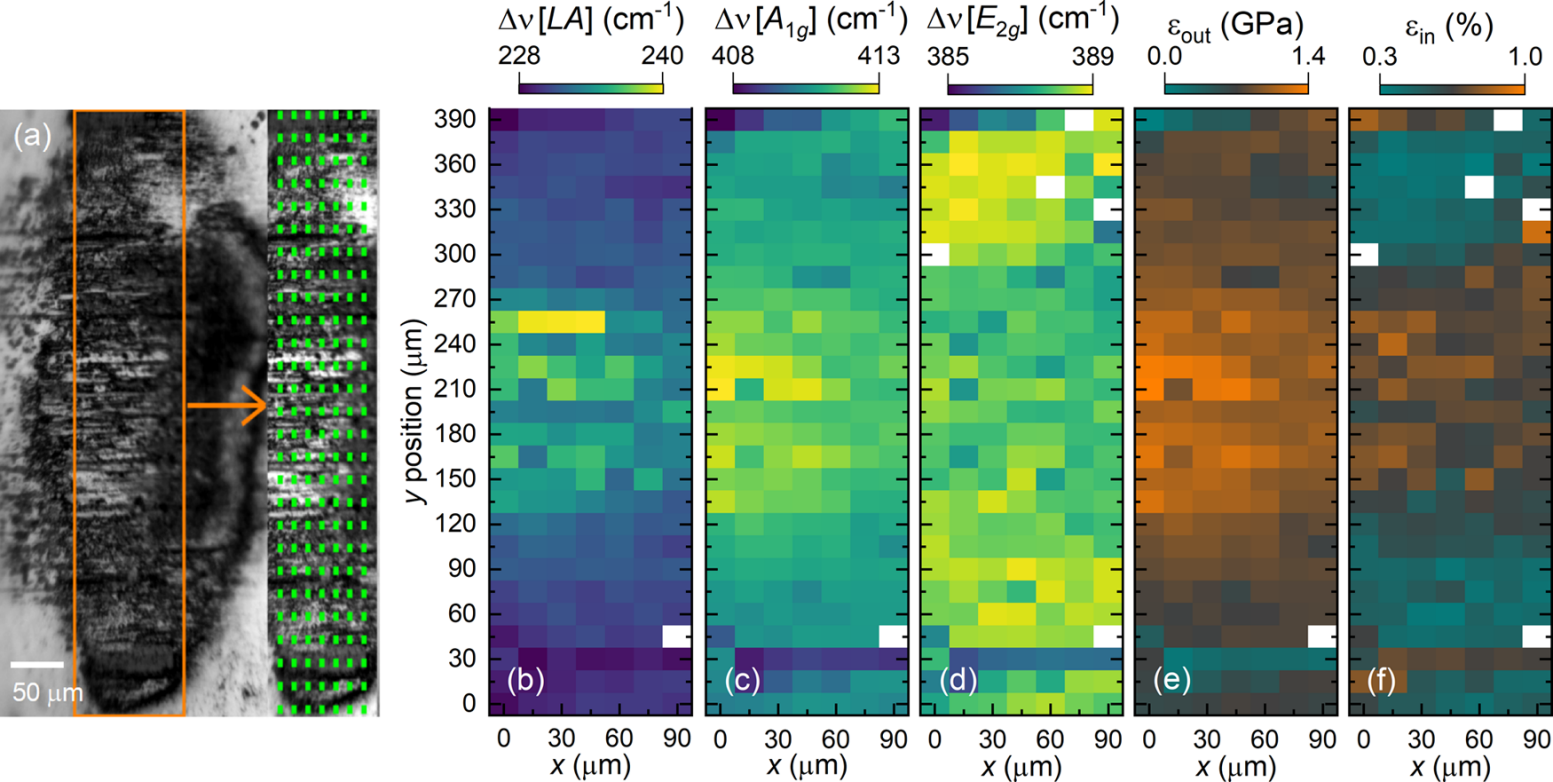
(a) Microscopic image of the tribological track at the steel counter body tested against MoS_2_:Cr (after 5000 rotations); the mapping area is marked by the orange frame, and the mapping points are illustrated by green squares. Spatial distribution of the Raman shift of the (b) LA(M), (c) A_1g_(Γ), and (d) E_2g_(Γ) modes. (e) Out-of-plane and (f) in-plane strain distribution; details on their calculations are given in the main text.

In this section, only the transfer material of the MoS_2_:Cr layer is considered as an example for the MoS_2_:X layers (except MoS_2_:V), since the strain distribution in the transfer material is comparable for the other modification elements, unless the layer is completely removed and only iron oxides are measurable. A microscopic image of a typical tribological contact area present at the steel counter body is shown in Figure 5(a). The spatial mapping points defined for the confocal Raman scattering are indicated by the green squares having an equal distance to each other of 15 μm along the *x* and *y* directions. The Raman shifts of the LA, A_1g_, and E_2g_ phonon modes detected at the counter body within the tribological contact area are shown in Figure 5(b)–(d). At the center of the area, the longitudinal acoustic phonon mode as well as the A_1g_ mode demonstrate shifts to high frequencies. Contrarily, the Raman shifts of the E_2g_ mode increase at the edges of the contact area, while at its center the mode possesses lower frequencies, as depicted in Figure 5(d).

The Raman shift Δ*ν* of the individual phonon modes is approximated by the expression . Here, *W* describes the coupling strength of the atoms involved in the phonon mode, *d* is the bond length, and *m* is the effective mass of the interacting atoms.^39,40^ Accordingly, pulling the atoms apart lengthens the chemical bonds compared to the relaxed atomic positions, and in turn, the frequency of the phonon mode decreases, while the coupling strength remains constant. Instead, a shortening of the chemical bond lengths in the atomic structure of the transfer material due to applied compressive stress enhances the Raman shift. Moreover, the geometry and spatial symmetry of the phonon modes allow for further specifying the type of induced strain. The frequency increase (blue shift/hardening) of the out-of-plane A_1g_ mode indicates compressive strain ε_out_ along the *c* axis of MoS_2_. On the contrary, the frequency reduction (red shift/softening) of the in-plane E_2g_ mode displays tensile strain ε_in_ in the *xy*-plane of the transfer material.

**Figure 6:**
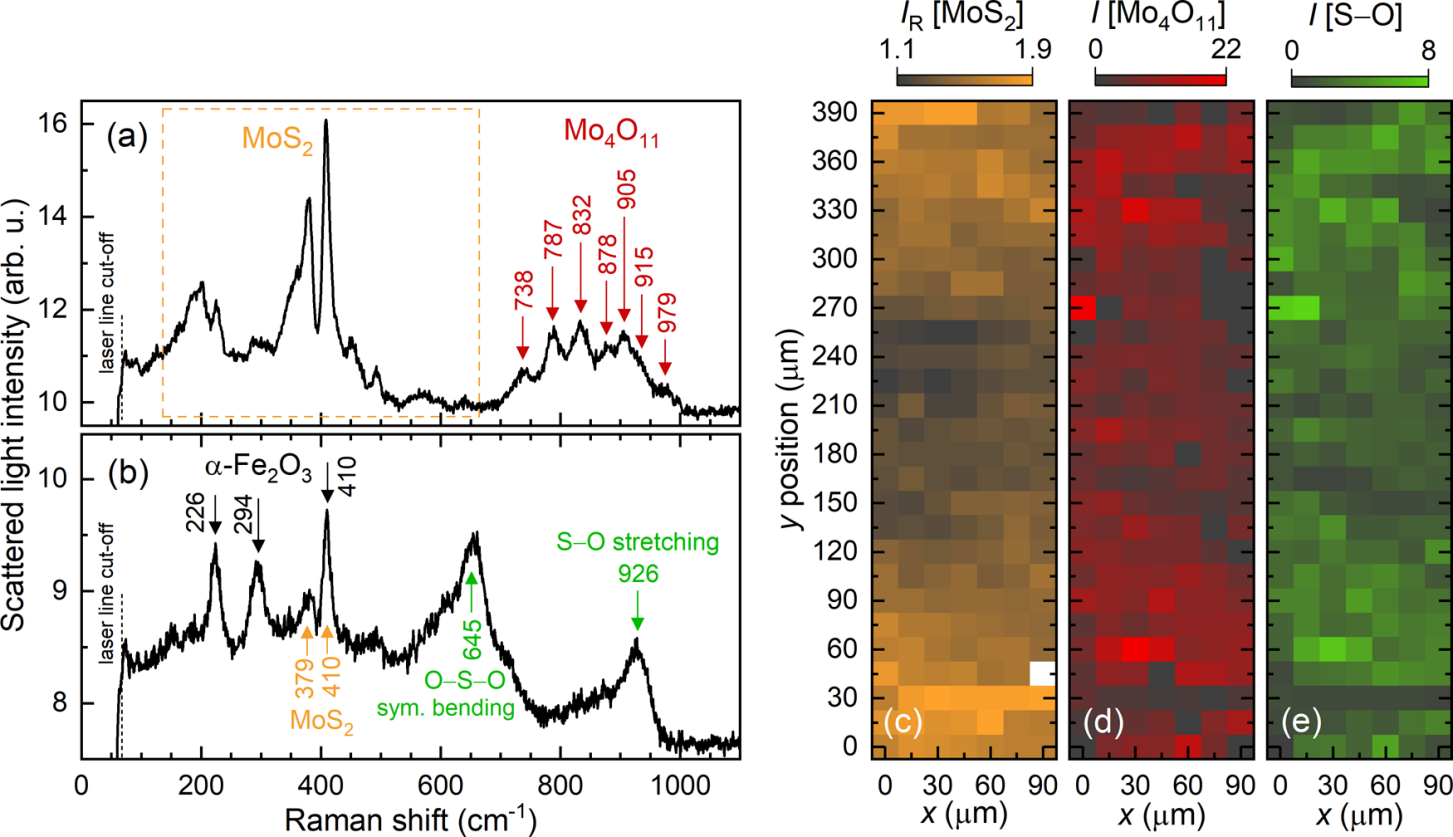
(a), (b) Raman spectra measured at the steel counter body tested against MoS_2_:Cu and MoS_2_:Zr, respectively, demonstrate Mo_4_O_11_-related and sulfur oxide Raman signals in addition to MoS_2_- and Fe_2_O_3_-related Raman lines. Similar results were obtained for the other counter bodies tested against MoS_2_:X (X = Cr, W, and Ti). Raman scattering mappings show (c) the intensity ratio *I*_R_, (d) integral intensity of the Mo_4_O_11_-related Raman band, and (e) the intensity of the S–O stretching mode.

In the following, we estimate the magnitudes of the compressive and tensile strain within the MoS_2_ transfer material. Compressive strain yields a hardening of the A_1g_ mode; whereby, the relative blue shift of the A_1g_ mode measured by 5.2 cm^–1^ corresponds maximally to ε_out_ = (1.4 ± 0.4) GPa.^41,42^ Following this relation, the spatial distribution of the compressive strain is given in Figure 5(e). The central tribological area of the counter body experienced the largest normal forces, leading to an irreversible compressive deformation of the MoS_2_ out-of-plane vibrations. While tensile strain hardly influences the A_1g_ mode,^43^ a significant pressure-induced Raman shift of the E_2g_ mode is relevant to calculate the tensile strain.^41,42^ In the literature, the Raman shifts range from −1.7 to −7.4 cm^–1^ per 1% tensile strain.^37,38,43^ We consider an average value of (−4.0 ± 0.8) cm^–1^ per ε_in_ = 1%. Using this relation, the mapping in Figure 5(f) displays weak tensile strain (about 0.3%) at the edges of the tribological contact, whereas at the center of the probed area the tensile strain rises to 1%. This strain evolution within the MoS_2_ transfer material is reflected by not only an increased separation between the *E*_2*g*_ and A_1g_ Raman lines but also a decrease in the intensity ratio *I*[A_1g_]/*I*[E_2g_], as illustrated in Figure 6(c). In agreement with ref (26), the intensity ratios are ≥1 which may be explained by a strong coupling of the A_1g_ mode to the excited *d*_*z*^2^_ electronic states of MoS_2_.^42^ As in the case of ref (44), the intensity ratio decreases as a function of rising tensile strain. Furthermore, the tensile strain breaks the local symmetry of the MoS_2_ transfer material which leads to a splitting into the two subbands E_2g_^–^ and E_2g_^+^. The E_2g_^–^ subband is observed as a shoulder at the low-energy flank of the E_2g_ Raman line. This splitting supports the in-plane tensile strain findings, as shown in Figure 5(f).

Moreover, the LA(M) mode is strongly shifted to high Raman shifts at the center of the ball-on-disk track. This mode is considered defect-induced so that its mode hardening is assigned to a reduction in the distance between defects (interdefect length) which, in turn, is probably due to the high compressive strain at the respective surface areas of the counter body.

### Spatial Distribution of Tribochemical Oxides

MoS_2_ Raman signatures were found in the transfer material at the counter body when it was tribologically tested against main bodies with MoS_2_:X (X = Cr, Cu, Zr, Ti, W) films. Moreover, the respective Raman spectra often contain lines related to molybdenum and sulfur oxides, as depicted in Figure 6(a),(b). In addition to the MoS_2_ Raman lines, in the spectral range from about 700 to 1000 cm^–1^ bands are recorded that originate from Mo_4_O_11_, a suboxide (Magnéli phase) of the stoichiometric MoO_3_.^25,45^ Furthermore, the O–S–O symmetric bending mode and S–O stretching mode are likely measured at 645 and 926 cm^–1^,^46^ respectively, as illustrated in Figure 6(b). To analyze the spatial distribution of these Mo and S oxides at the tribological contact area of the counter body, their Raman band intensities are approximated: a broad Gaussian function is used to describe the Mo_4_O_11_ signatures, and a narrow Gaussian function is fit to the S–O mode. The corresponding intensities are depicted in Figure 6(d),(e). The Raman signatures of Mo_4_O_11_ and sulfur oxide are most pronounced at the edges of the contact area, while at the center they are only weakly observed.

The substitution of sulfur by oxygen leading to the formation of Mo_4_O_11_ is most probably thermally induced: it was shown that the heating of a MoS_2_ crystal or a MoS_2_ lubrication film in an oxygen-rich atmosphere leads to the formation of different molybdenum oxides, in particular for temperatures between 330 and 400 °C.^47−49^ It is astonishing that only Mo_4_O_11_ is probed by confocal Raman scattering, while other oxides like MoO_2_ or MoO_3_ are not identified. The formation of Mo_4_O_11_ has a Gibbs energy of Δ_f_*G*^0^ = −2523.1 kJ/mol which is significantly lower than Gibbs formation energies of MoO_2_ (Δ_f_*G*^0^ = −532.1 kJ/mol) and that of MoO_3_ (Δ_f_*G*^0^ = −667.5 kJ/mol).^50^ Thus, from a thermodynamic point of view, the formation of Mo_4_O_11_ occurs more likely than that of MoO_2_ and MoO_3_. Even higher oxygen states (like Mo_8_O_23_ or Mo_9_O_26_) are kinetically inhibited and are mechanically unstable due to their large spatial dimensions. The presence of Mo_4_O_11_ which is a Magnéli-phase of MoO_3_ is tribologically beneficial, since it allows for the creation of a layered structure similar to graphite and MoS_2_ as well as it is chemically stable at high temperatures.^51,52^

The spatial distribution of Mo_4_O_11_ mainly at the edges of the tribological contact may be explained by a mechanical displacement during the ball-on-disk test, resulting in a material accumulation at the contact edges. Moreover, the edges are exposed more frequently to the air atmosphere so that the environmental oxygen required for the tribo-oxidation may reach the MoS_2_ transfer material more easily. In that context, we however refrain from estimating local temperatures which are necessary to initiate the tribo-oxidation processes, since they depend both on the temperature as well as on the crystallite size of MoS_2_ and the spatial dimension of the MoS_2_ wear particles. Besides that, we omit to propose a formation mechanism of the sulfur oxide phonon modes spectroscopically observed within the MoS_2_ film. On the one hand, Gibbs energy data are available only for gaseous phases—to the best of our knowledge, and on the other hand, the tribologically time-variant integration of oxygen into the Mo–S–O network forming stretching and bending modes with sulfur ions was not studied.

**Figure 7:**
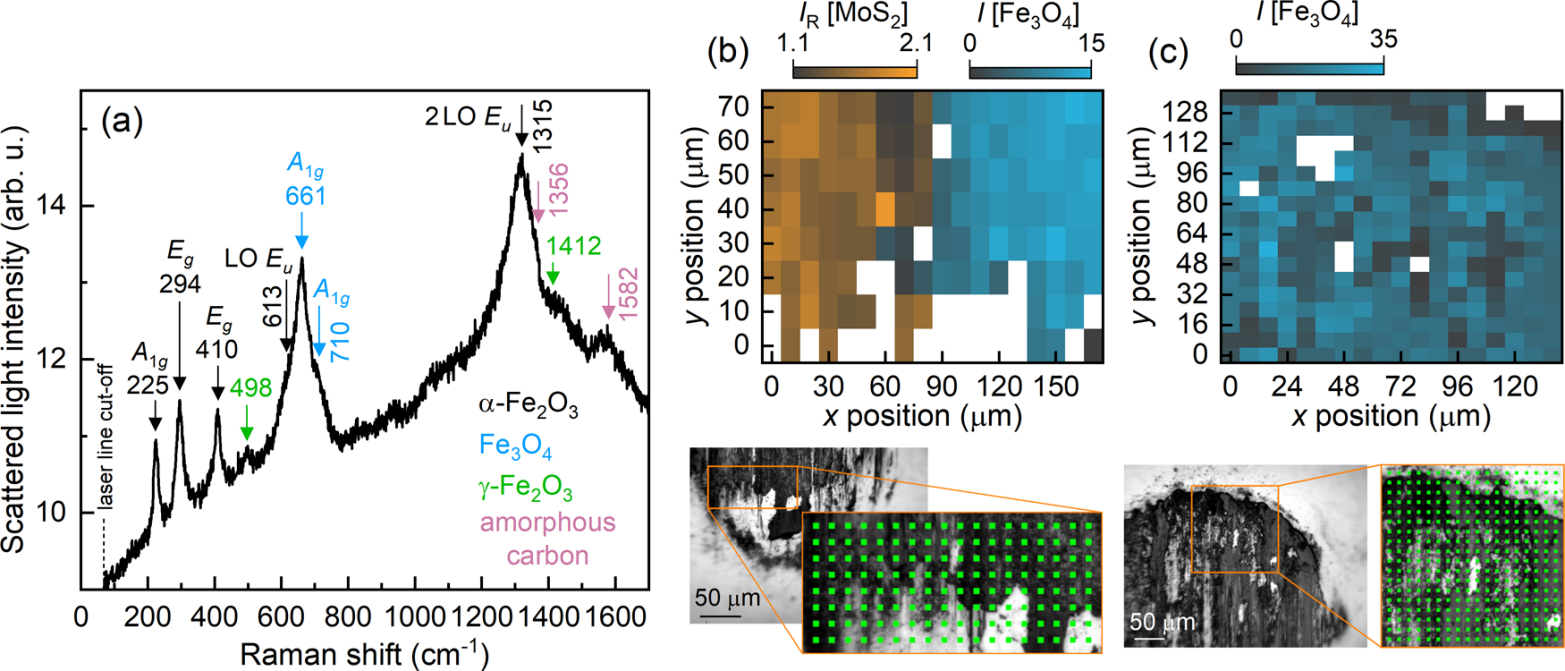
(a) Raman spectrum showing Fe_2_O_3_ and Fe_3_O_4_ lines measured within the central area of the tribological track at the counter body surface which was tested against a MoS_2_:V film. Microscopic image and Raman scattering mapping at the counter body tested against a (b) MoS_2_:Zr and (c) MoS_2_:V main body. The intensity of the significant magnetite Raman peak at about 661 cm^–1^ is taken as an indicator for oxidized iron compounds.

The tribological ball-on-disk tests using main bodies with MoS_2_:V and MoS_2_:Zr result in a specific composition of the tribological material at the steel counter-body surfaces. As shown in Figure 7(a), clear Raman signatures attributed to iron oxides are observed. In addition to the peak at about 661 cm^–1^, the peaks at 225, 294, 410, and 1315 cm^–1^ are especially pronounced. The latter four Raman lines most probably originate from hematite (α-Fe_2_O_3_) and belong to the A_1g_, E_g_, E_g_, and 2E_u_ vibrations, respectively.^53−55^

The longitudinal-optical (LO) E_u_ mode of α-Fe_2_O_3_ at about 613 cm^–1^ is rather weak in comparison with the aforementioned phonon modes. The LO E_u_ mode does not solely explain the intense and broad peak at 661 cm^–1^. Magnetite Fe_3_O_4_ leads to a characteristic and intense Raman band at about 667 cm^–1^^55^ with a shoulder at about 710 cm^–1^. Furthermore, weak signals stemming from maghemite (γ-Fe_2_O_3_) are detected at 498 and 1412 cm^–1^.^53−56^ While the α-Fe_2_O_3_ related Raman lines are observed only at heavily worn and rough surface areas, the Fe_3_O_4_ Raman signature at 661 cm^–1^ is typically detected at the counter body tribologically tested with MoS_2_:V and MoS_2_:Zr coated main bodes.

To analyze the spatial distribution of the iron oxidation with regard to the MoS_2_ transfer material at the steel counter body, the 661 cm^–1^ magnetite band intensity *I*[Fe_3_O_4_] which is derived from a Gaussian fitting is depicted in comparison to the MoS_2_ intensity ratio *I*[A_1g_]/*I*[E_2g_] in Figure 7(b),(c). These Raman mappings are gathered from the tribological contact areas at the steel balls (see microscope images at the bottom of the panels), which were run against a MoS_2_:Zr film, Figure 7(b), and a MoS_2_:V film, Figure 7 (c). As shown in panel (b), the MoS_2_-related intensity ratio decreases significantly toward the center of the contact area, where the intensity of the magnetite Raman signature becomes pronounced. The spatial transition from MoS_2_ at the contact area edges to Fe_3_O_4_ at the center occurs within a range of 30 μm. On the contrary, at the counter body tested against MoS_2_:V, MoS_2_ transfer material is not detected; instead, the worn counter bodies’ surface exhibits Raman scattering signals only from iron oxides. It is worthwhile to mention that the steel ball surface regions adjacent to the contact area do not demonstrate any oxidation; hence, thermal diffusion and strain fields over tens of micrometer distances promoting the oxidation of steel (AISI 52100) are ruled out.

The appearance of, in particular, magnetite at the counter body surface is due to its direct contact with surface positions at the main body where the MoS_2_ film was not present anymore, and in turn, the steel substrate (16MnCr5) was exposed. Within the steel–steel contact, thermal and high mechanical energies are present, thus providing a high probability for the oxidation of Fe. The steel–steel contact situation itself during the tribological testing, as discussed for the MoS_2_:V film within the frame of Figure 3(i),(j), is mainly attributed to increased adsorption and incorporation of water molecules into the MoS_2_:V film leading to significant friction and wear. The spatial distribution of MoS_2_ and Fe_3_O_4_, for the MoS_2_:Zr case shown in Figure 7(b), in combination with the low wear coefficient, indicates that the local temperature and stress also play an important role. The highest pressure prevails at the center of the contact area, thus making a removal of the MoS_2_ material more probable. This is evidenced by the micrometer-resolved observation of magnetite at the contact center, while at the edges, MoS_2_ transfer material is agglomerated. The presence of mainly magnetite leads us to the assumption that the local temperatures were only moderate within the tribological contact. The thermal transformation of maghemite and magnetite to hematite is activated by temperatures ranging between 370 and 600 °C being sensitive to the crystal size.^57^ Additionally, the oxygen partial pressure^58^ and applied pressure^59^ affect the transformation process. Accordingly, we propose that the local temperatures were below 370 °C. Furthermore, Raman signals characteristic for zirconium oxides are not detected within the tribological tracks of the main and counter body. Raman lines of, e.g., ZrO_2_ may be broadened^60^ and may be overlapped by the MoS_2_ and Fe_3_O_4_ Raman lines. Their absence in the Raman spectra measured is in agreement with the general observation that the transition metal ions used for the MoS_2_ film modifications are not oxidized most probably due to their low concentration (about 20 at. %) and/or the sensitivity limitation of our Raman scattering spectroscopy setup.

### Tribologically Induced Formation of Few-Layer Graphene

Besides the tribologically induced formation of sulfur and molybdenum oxides, a further self-lubricating phenomenon is governed by the formation of amorphous carbon (a-C) and, in particular, few-layer graphene at the steel counter bodies. In the Raman scattering spectrum shown in Figure 7(a), the broad signals at about 1356 and 1582 cm^–1^ resemble the D and G Raman peaks of *sp*^2^ hybridized amorphous carbon characterized by a low fraction of *sp*^3^ hybridized carbon bondings. The hybridized carbon bondings and, in turn, the amorphous carbon are formed, on the one hand, due to the exposition of the steel substrates and, in turn, due to the continuous tribological load leading to free carbon atoms originating from the steel surfaces. On the other hand, the surfaces may be contaminated adventitiously by hydrocarbons from the ambient air adsorbed on the MoS_2_.^61−63^

**Figure 8:**
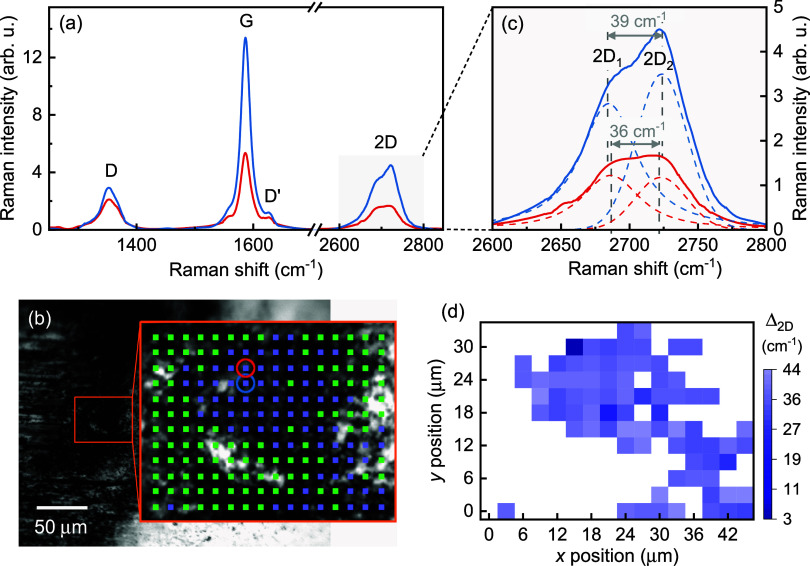
(a) Raman spectra of few-layer graphene detected within the tribological contact at a steel (AISI 52100) counter body tested against MoS_2_:Cr. (b) Microscope image of a part of the tribological contact; the area used for Raman mapping is indicated by colored squares, whereby the blue color indicates the presence of few-layer graphene. The 2D band splitting shown in detail in (c) is spatially analyzed by a Raman mapping depicted in (d). The *x* and *y* positions correspond to those of the points sketched in (b).

Surprisingly, at some positions of the counter bodies which were tested against Cr-modified MoS_2_ films, sharp D and G peaks as well as the second-order 2D peak are observed. Figure 8(a) shows exemplary Raman spectra recorded at the counter-body surface positions marked by color-coded circles in the microscopic image in Figure 8(b). Furthermore, the 2D band is split significantly, as clearly depicted in Figure 8(c). Here, the positions of the 2D_1_ and 2D_2_ peaks are evaluated from Voigt function fittings; see dashed lines. For both spectra, the splitting Δ_2D_ amounts to 36 and 39 cm^–1^, respectively. The mapping which is illustrated in panel (b) reveals variations of the 2D band splitting from Δ_2D_ = 2.7 to 43.8 cm^–1^. The mean value of the 2D splitting is given by (35.9 ± 5.3) cm^–1^.

The splitting of the 2D Raman band depends on either the number of layers of graphene^64^ or uniaxial strain applied to graphene.^65^ Since strain should also lead to a significant splitting of the G peak, which is however not observed, we assume that different numbers of graphene layers were formed within the tribological contact at the steel counter body. Based on the splittings as well as the correlation between an intensity enhancement of the G peak and an increase in the number of graphene,^66^ between 6 and 23 layers of graphene were presumably formed. Due to the appearance of the D’ peak and a slight broadening of the D as well as G peaks the graphene layers are subjected to disorder.^64,67^ Disordered graphite may also be present. Carbon atoms likely from the steels (main and/or counter body) and/or ambient air establish amorphous carbon flakes within the tribological contact due to the thermo-mechanical load, while the tribologically induced few-layer graphene flakes are highly ordered. We assume that the formation of highly ordered graphene flakes on the counter body is possible due to the low friction and wear so that the thermo-mechanically energetic input is sufficiently weak leading to a self-organized network of few-layer graphene. Instead, the vanadium modification of MoS_2_ results in high friction and wear, as well as a pronounced film abrasion; this harsh tribological condition gives rise to the formation of only iron oxides. Details on the thermal and mechanical energies being necessary for the graphene formation as well as beneficial wear and friction effects of the few-layer graphene flakes shall be analyzed in future studies.

## Conclusions

Confocal Raman scattering spectroscopy with micrometer spatial resolution reveals structural and tribochemical changes in the MoS_2_-based transfer material at the steel counter bodies tribologically tested against differently modified MoS_2_:X films.

**Figure 9:**
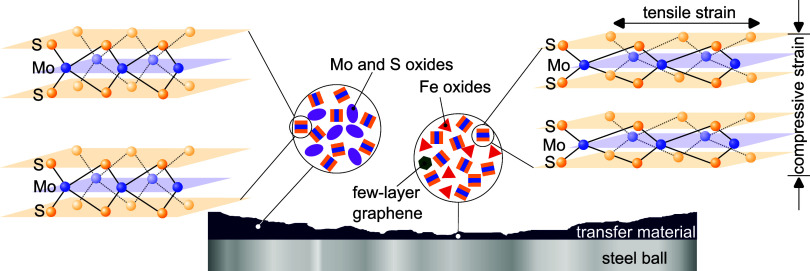
Schematic overview of the strain, oxidation, and graphene formation within the MoS_2_-based transfer material at the steel (AISI 52100) counter body surface.

The E_2g_ and A_1g_ Raman lines are more spectrally separated from each other at the center of the tribological track than at the edges. It indicates variations in the tensile and compressive strain within the MoS_2_ transfer material, as schematically shown in Figure 9. The compressive strain amounts maximally to ε_out_ = (1.4 ± 0.4) GPa, while the relative tensile strain varies between ε_in_ = 0.3 and 1%. Additionally, the low ratio between the A_1g_ and E_2g_ line intensities, for the MoS_2_ transfer material, shows a structural degradation as a consequence of a realignment of MoS_2_ grains from an out-of-plane to an in-plane orientation. It is observed mainly for the counter bodies run against MoS_2_:(Zr, Cr) films. Interestingly, for the case of MoS_2_:Cu, the out-of-plane vibration A_1g_ is more disturbed at the worn surface areas of the main body than at the counter body. It hints at a copper-characteristic cladding effect and its weak shear strength, thus allowing for structural realignments.

A decrease in the MoS_2_ Raman line intensities is accompanied by an intensity enhancement of sulfur and molybdenum oxide vibrations, which are particularly agglomerated at the edges of the tribological track. The formation of these tribo-oxidation products weakly depends on the type of modification elements. For all cases, an oxidation of a modification element itself is not detected. In addition to that, Fe_3_O_4_ and α- and γ-Fe_2_O_3_ are Raman spectroscopically detected within the tribological tracks, when the MoS_2_:X film is removed and MoS_2_-based transfer material is absent at the counter body, i.e., when the substrate runs against the steel sphere as well as wear and friction are high. The iron oxidation occurs especially for MoS_2_:V and partially (at the center of the tribo-track) for MoS_2_:Zr. The failure of the MoS_*x*_:V film may be ascribed to the water affinity of vanadium so that the film and wear particles become brittle, leading to high friction. All other element-modified MoS_2_ films exhibit improved friction properties, probably due to the densification of the microstructure and the establishment of a (002) basal-plane orientation. Furthermore, few-layer graphene subjected to disorder is formed within the tribological contact between MoS_2_:Cr and the steel sphere revealed by sharp D and G Raman lines and specific splittings of the 2D Raman band at about 2700 cm^–1^. The splitting values allow us to estimate the number of graphene layers, varying between 6 and 23 layers. These multiple graphene layers are detected at the edges of the counter bodies’ tribological track.

The spectroscopic access to the structural and chemical MoS_2_ features of the tribological system including an element-modified MoS_2_ main and in particular a steel counter body allows for evaluating the functionality of MoS_2_ as solid lubricant and for predicting material failure already at the microscopic level.
